# The up-down-up pandemic news experience: A mixed-method approach to its negative and positive effects on psychological wellbeing

**DOI:** 10.1177/14648849221135137

**Published:** 2022-11-21

**Authors:** An Nguyen, Antje Glück, Daniel Jackson

**Affiliations:** Faculty of Media and Communication, 6657 Bournemouth University, Poole, UK

**Keywords:** pandemic news, news avoidance, positive news, constructive news, pandemic news, mental health, emotional effects

## Abstract

Existing research has documented the dynamics of increased news consumption alongside – paradoxically – increased news avoidance during the Covid-19 pandemic, highlighting its adverse effects on mental health and emotional wellbeing. However, for methodological and theoretical reasons, research still lacks specifics on what types of negative psychological responses were directly triggered by pandemic news, how prevalent they were in the population, how they manifested in daily life, and what could be the alternatives to them. Further, the almost exclusive focus on negative effects has led to a relative negligence of the positive sides of pandemic news. This study takes a mixed-method approach to address these gaps, combining 59 interviews and a follow-up survey with a representative sample of 2,015 adults across the UK. We found that pandemic news consumption, driven primarily by the need for personalised surveillance in an uncertain situation, oscillated in parallel with its severity and associated lockdown restrictions. The influx of repetitive bad news triggered many negative feelings besides general pandemic anxiety – namely fear, despair and moral outage (anger and disgust). This led to various alterations of daily routines, including news avoidance. Such adverse effects were offset by the reassurance, happiness and hope that the news did, at least occasionally, brought to audiences during the pandemic. Participants suggested several potential “good news” categories that point to the need for constructive news forms that not only inform but also inspire, motivate and/or empower people in personal or collective ways.

## Introduction

The manifold disruptions caused by Covid-19 have affected news consumption in ways not seen before. As people turned to the media to gratify cognitive, affective and socially integrative needs amidst the sheer disruption of the pandemic, early research has documented the ambivalence between the need to monitor an evolving crisis and the protection of mental and emotional health, which led to a rapid fluctuation of news consumption (Broersma and Swart, 2021; de Bruin et al., 2021; Groot-Kormelink and Klein-Gunnewiek, 2021; Skovsgaard and Andersen, 2020; Van Aelst et al., 2021). News avoidance attributed to the negative effects of pandemic news on psychological wellbeing has emerged as a key issue of concern for scholars. Most research, however, has focused on such effects at the expense of attention to positive aspects of Covid-19 news consumption. Among those focusing on the adverse effects, there is still a lack of specificity on the kind of negative feelings caused by pandemic news, how prevalent they were in the population, and how they manifested in the daily life of lockdown publics.

This study thus takes a more nuanced approach to add depth to current knowledge of pandemic news consumption and its effects on psychological wellbeing. In seeking to explain the fluctuations of Covid-19 news consumption over a one-year period of three national lockdowns, it employs a mixed-method examination of both negative and positive dimensions in the relationship between personal surveillance of pandemic news, mental and emotional states, and news avoidance. Based on 59 in-depth interviews and a survey with 2015 citizens across the UK, we examine and interlink three different foci: (a) the pervasive negative news environment’s effects on users’ psychological wellbeing, with a focus on the specific characteristics of news-induced feelings and behaviours (including multiple forms of news avoidance); (b) the positive psychological effects of pandemic news consumption and their potential contribution to mitigating news avoidance; and (c) alternative types of pandemic news that users perceived as helpful for them to offset its adverse effects.

## Research into the COVID-19 pandemic news experience

News, as an uncertainty reduction means, is often considered to be a lifeline in times of crises – and the pandemic is no exception. In the UK’s first lockdown week in March 2020, for example, virtually every online user (99%) accessed news at least once a day (Ofcom 2020) – a fact that might be deemed almost fiction-like by normal measures of news consumption. After the initial steep surge in news consumption, however, research found an increasingly heterogenous approach, with the surge losing ground to a decline in news use and a fast increase in news avoidance, especially among women and younger people (Villi et al., 2021; Ytre-Arne and Moe, 2021).

Within these rapid changes is consumers’ reconfiguration of how they consume the news to deal with the pandemic and its disruptions. Here, the theoretical framework by Trenz et al. (2021) can serve as a useful starting point. As news users are forced into social isolation during lockdowns and exposed to a monothematic news agenda, they experience high levels of uncertainty and stress and respond to them with two seemingly contradictory coping strategies, namely news seeking and news avoidance.

The first takes the form of an information-utility approach, in which users implement an active information-oriented search strategy to survey the environment, to make sense of the unexpected crisis, and to grasp what public health measures are in place for them to follow. All this is to serve the purposes of reducing uncertainty (De Bruin et al., 2021) and/or improving one’s social wellbeing through accessing a shared crisis experience – i.e., feelings of solidarity and “security in the form of bonds, affiliation and shared destiny” (Trenz et al., 2021: 10). During the pandemic, for instance, rituals such as governmental press conferences and the “liveness” of events enabled the perception of a shared experience. Frequent news users and “news junkies” were more likely to benefit, as heavier news consumption facilitated a stronger social tie and more active participation in public debates (Broersma and Swart, 2021). Pandemic news seeking led consumers to reconfigure their news intake, revising old routines and/or forming new habits (ibid). In the early days of the pandemic, for instance, as people sought news to reduce uncertainty and gain orientation, many turned to places that they would have not before – such as "alternative" news or social media platforms (Newman et al., 2020). Trenz et al. (2021) see these new habits as part of a resilience-enhancement strategy.

Such information-utility approach, however, might work in the opposite manner: instead of helping users to cope with stresses, news could generate more stress among users (Broersma and Swart, 2021). In the US, for example, more than four in ten reported “feeling worse emotionally” from watching Covid news (Pew Research Center, 2020). In Germany, a survey found that three exposure measures – frequency, duration and diversity – were positively associated with symptoms of depression, Covid-specific and Covid-unspecific anxiety (Bendau et al., 2020). Further, people with pre-existing anxieties tended to be experiencing more distress from the news (ibid). Similarly, a survey with people aged 13–25 with a history of problematic mental health by YoungMinds UK (2020) found the vast majority (83%) reported the pandemic worsening their mental health, with news use being the most frequently cited unhelpful activity (66%). The literature points to two reasons for this. First, news consumption itself introduces and reintroduces uncertainty due to the presence of knowledge gaps, incomplete solutions, and expert disagreements in the media (Trenz et al., 2021). Second, intense news seeking leads users into an information overload that generates multiple forms of informational and emotional overload, or news fatigue (de Bruin et al., 2021; Groot-Kormelink and Klein-Gunnewiek, 2021; Ytre-Arne and Moe, 2021).

These factors converge to give rise to the second practice of resilience, which is the opposite of news seeking: news avoidance. Here, limiting or abandoning exposure to news – either for a short or long term – helps users to achieve resilience through two closely related processes: (i) avoidance can lower the chance for users to experience the distress and anxiety caused by uncertainty; and (ii) avoidance can help users to escape the subjective experience of bad feelings caused by the predominantly negative nature of Covid news. This is, of course, not peculiar to the pandemic: the abundance of negative news has long been a key driver of worrying news avoidance in Western countries (Constructive Institute, 2020; Gottfried, 2020; Serrano-Puche, 2020; Shehata et al., 2015), particularly among women and younger people (Toff and Kalogeropoulos, 2020; Villi et al., 2021). But, as Trenz et al. (2021) argue, the pandemic is like no previous situation: being exposed to a ceaseless influx of bad news about a single issue, in an enforced social isolation, could create unparalleled levels of negative mental and emotional effects, thus strengthening the need to switch off.

The link between pandemic news avoidance and its negative psychological effects has been established across the world. In the UK, two-thirds of those who avoided Covid-19 news cited its “bad effect on my mood” and 33% the feeling that “there is too much news” as reasons (Kalogeropoupos et al., 2020). In the Netherlands, Groot-Kormelink and Klein-Gunnewiek (2021) found that young audiences’ news consumption in the early phase followed four distinctive stages – *indifference* (due to the perception of the pandemic being far away); *shock* (and, consequently, a huge spike in news consumption) as the outbreak arrived; *Corona fatigue* (an informational and emotional overload) as the virus advanced; and the becoming of a *new normal*. Of these, they found shock and fatigue to be the most critical: they demanded users to fundamentally adapt news habits – including a reduced or more controlled consumption of news – so that they were informed without letting their news intake overwhelm their information- and emotion-processing capacities. In a Dutch survey, de Bruin et al. (2021:1) found that, while mental wellbeing did not engender news avoidance, news avoidance had a slight positive effect on mental wellbeing, meaning that “avoiding news is sometimes necessary to stay mentally healthy”.

From Australia, Mannell and Meese (2022, p. 314) found news users striving to balance between “two competing needs: the need to mitigate the negative impacts of news consumption, and the need to keep up to date with important developments about the lockdown”. The tension between them leads to what the authors labelled as *situated* news avoidance (as opposed to habitual news avoidance). In Germany, Bendau et al. (2020) found that those spending more time on Covid-19 news felt a more pronounced need to reduce it to avoid its negative effects on mental health. Perhaps, as they argue, consumers see overconsumption of news and information as a risk factor for mental illness in the crisis. The risk of pandemic news consumption has also gone beyond the media and communication realm to become a serious concern for health professionals and organisations. As the pandemic deepened, for instance, MentalHealth.org, a major health charity in the UK, advised its followers to “find a balance” in news consumption to reduce stress. Likewise, the National Health Service recommended its users to “limit the time [you] spend watching” news and to “(turn) off breaking-news alerts on your phone” (NHS, 12 May 2021).

In sum, pandemic news consumption oscillates between phases of intensity and laxity and between news seeking and news avoidance, and this has much to do with its negative effects on users’ psychological wellbeing. Several things, however, require more attention before we reach a balanced, more nuanced understanding of what pandemic news does to the audiences’ mental and emotional state.

First, the evidence base for the negative effects of pandemic news on psychological wellbeing needs to be expanded in several aspects. For one thing, most research so far tends to describe such effects through catch-all terms – such as ill mental health, emotional drain and bad mood – but lacks details on specific feelings. Often, descriptions do not go far beyond common mental and emotional states that the pandemic-wounded public would have experienced, with or without news consumption, such as despair, anxiety and fear. Other possible negative feelings directly triggered by news per se – e.g., moral outrage (such as anger) or pity towards what is reported in the news – have not been studied in detail. For another, the vast majority of studies approach the negative psychological effects of pandemic news from a qualitative perspective, stopping at describing their existence in small and non-representative samples, with no external validity to generalise their magnitude in the broader population. Even the contribution of negative news-induced emotions to news avoidance has been established mainly through interpretation of qualitative interviews rather than through rigorous statistical testing, except for Bendau et al. (2020) and De Bruin et al. (2021). This study thus takes a step further by combining qualitative and quantitative data to seek both depth and breadth in answering the following:

RQ1 **What were the most prevalent negative psychological responses to pandemic** **news and how did they contribute to its avoidance in the UK population?**

Second, as scholars have focused on the negative psychological effects of Covid-19 news, they tend to overlook the other side of the coin. If news seeking is a resilience strategy in times of crisis, then it must have some positive effects on users’ mental and emotional state. However dark the pandemic situation was, for instance, some people might at least sometimes find news a source of joy, assurance or hope for very personal reasons. Further, it is reasonable to expect that such positive reception might reduce users’ tendency to reduce or avoid pandemic news. So far, however, there is little evidence of this. We ask:

RQ2 **What were the most prevalent positive psychological responses to pandemic** **news and how did they relate to news avoidance in the UK population?**

Third, although the abundance of negative Covid-19 news has been identified as a key cause of news avoidance, no study has asked audiences what they think could be the alternatives. Given that the pandemic was a pro-longed catastrophic crisis, what type of “good news” did audiences think the media could provide to offset the abundance of bad news? We ask:

RQ3 **In what ways did UK audiences think the media could make pandemic news** **more positive?**

## Method

This study is part of a larger project that investigated how UK audiences experienced Covid-19 news and what they expected the media to do to help local communities recover from the pandemic. The data came from the early stage of the project, which combined in-depth interviews and a survey with UK news users.

### In-depth interviews.

59 members of the public were interviewed between 15 February and 3 March 2021. The sample was recruited by a professional research firm, to ensure proportionate representation relative to the UK population in terms of *gender* (49% males and 51% females), *age groups* (19% in 16–25 group, 22% in 26–35, 19% in 35–45, 15% in 46–55, 15% in 56%–65% and 10% in 66+); *ethnicity* (10% from minority groups); and *regions* (Scotland, Wales, Northern Ireland and a good spread of England, including London, Southeast, South West, East Midlands, West Midlands, North West, North East, and Yorkshire). As one aim of the broader project was to explore how the news could assist local communities to recover from the pandemic, we intentionally included 20 “community leaders” in the sample. These were basically ordinary citizens who were playing or had played some formal or informal role in communities – e.g. support workers, mother and baby group leaders, Brownie/scout leaders, school governors, community sports coaches, local club committee members, local business leaders etc.

Interviews lasted 45–60 min and were conducted over Zoom. Each was semi-structured in three sections: (a) general news consumption in pre-pandemic times; (b) pandemic news use experience and expectations; and (c) the psychological empowerment potential of solutions journalism (SOJO) in general and in the context of the pandemic. Participants were not told about these specific topics in advance. For this paper, we use data from the first two sections, when participants had not been introduced to the SOJO concept and therefore were not potentially primed towards it in their responses.

### Survey

The findings of the interviews were fed into the design of a national survey on pandemic news experiences. The questionnaire gathered information to quantify and scale the phenomena observed in the interviews, especially the psychological effects that emerged frequently in the interviews, as well as to explore other relevant issues that have been raised in the literature on crisis news consumption. Respondents were specifically asked about, *inter alia*, their uses of Covid-19 news, its impact on mental and emotional states, how it shaped their pandemic experience and what they expected from journalism in the exit from the pandemic.

The survey was administered by Opinium, a professional research firm. Opinium recruited participants from a panel of about 40,000 UK citizens who signed up to take part in its surveys on a range of subjects in exchange for small incentives. Participants were not told about the survey’s subject when they were invited to prevent response bias. The final sample included 2015 qualified respondents, which were demographically representative of the UK’s adult population.

It is noteworthy that both qualitative and quantitative data were collected after the UK had gone through three national lockdowns and various local ones. The survey itself was live from 22 to 24 March 2021 – exactly 1 year after the UK went into the first lockdown. This is a crucial difference from previous research, which has examined pandemic news consumption within early single waves of the virus outbreak rather than across different waves. As De Bruin et al. (2021) argued, a full picture must include how news uses and avoidances evolved *throughout* the pandemic. While we did not collect time-series data, this study did specifically ask audiences to reflect on how their news uses changed across the waves. Inevitably, all self-reported data like these depend on audiences’ ability to recall. As such, all interviewers were trained to use probing or clarifying questions wherever necessary to encourage deep reflections on the chronological order of news consumption changes since the first lockdown. During data analysis, we cross-checked and eliminated any actual or potential inconsistencies in interviewees’ responses. Overall, despite their subjective nature, we are confident that the interview data had reached a very high level of authenticity and trustworthiness.

### Data analysis

Audio recordings of interview data were transcribed verbatim and analysed in NVivo using a process of inductive thematic analysis. This provided for the contextual development of key instances and themes across the transcripts grounded in existing literature. Colleagues within the wider project team acted as “critical friends” in the analytic process, providing reflective “sound boards” as multiple themes were identified and contextualised. All interviewees’ names here are pseudonyms. As for survey data, this paper will use descriptive analysis to scale-up what we found in the in-depth interviews. All percentages reported below would have a sampling error of ±2%. For the relationships between news avoidance and its negative and positive psychological effects, we conducted multivariate regression analyses with statistical controlling for age and gender (more below).

## Findings

Previous research found a clear oscillation in news consumption within the first wave of the pandemic. With the usual caveat about the nature of self-reported data, we found that audiences’ perceived changes in news consumption across multiple waves followed a similar “inverted bell curve” pattern. In general, participants reported higher levels of news consumption – both in terms of use frequency and time spent – than pre-pandemic times, with some developing new habits (e.g., heavier use of news apps or more tolerance of data and statistics). Following a steep increase in the first lockdown (March 2020), however, engagement with news reduced along with easing restrictions over the summer months, and then rose again, to some extent, in the autumn and winter, when rising infection cases and deaths forced the UK into lockdowns again.

Some participants attributed their up-down-up pattern to the extra amount of free time afforded by lockdowns. For the most part, however, they linked it to what we would call the drive for *personalised surveillance* – i.e. the overwhelming lockdown-induced need to monitor the pandemic *in relation to* how it affected their own daily routines and concerns. As people sought to understand the evolving situation, detailed local/national statistics – especially transmission rates, hospital admissions, deaths and, later, vaccination rates – and updates on lockdown restrictions were in high demand. Even when they looked beyond local/national contexts, it was often about places with which they have personal connections. Due to this personalised surveillance drive, news consumption went up and down in parallel with the severity of the pandemic and its alternate waves. Embedded in this process are the contradictory effects of surveillance on users’ psychological wellbeing.

### “A barbed wire” of bad news to avoid: negative effects of pandemic news on psychological wellbeing (RQ1)

Heavy news consumption out of the personalised surveillance drive in an uncertain, rapidly evolving situation took a heavy toll on the psychological wellbeing of many participants. First, for some interviewees, news per se could not offer them a sense of control, especially during the early days, when nobody could be certain about anything. Second, in line with previous research, there was the pervasive feeling of information overload. Interviewees frequently lamented about fatigue caused by the sheer volume and intensity of, to use one interviewee’s word, “samey” news about Covid-19. This fatigue was worsened by the very negative focus of Covid-19 news: all but a few interviewees felt that while repeated exposure to the same news was bad, it was repeated exposure to the same bad news – especially the influx of case numbers, death statistics and lockdown uncertainties – that triggered much worse psychological distress. One participant, Nikki, compared this to a barbed wire around her daily life:

You just heard from the five news articles exactly the same thing [and you wanted to shout], “Please shut up and leave me alone, I don’t feel like it.” … So, [it’s like] you put a barbed wire around and really can't move.

**Figure 1.**
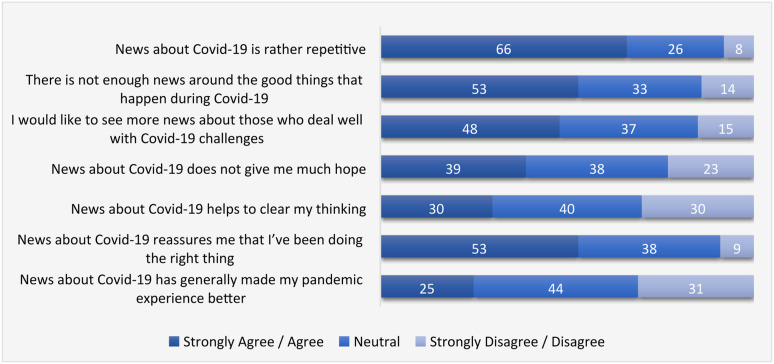
UK audiences’ reflections on their general pandemic news experience (%, n = 2015).

**Figure 2.**
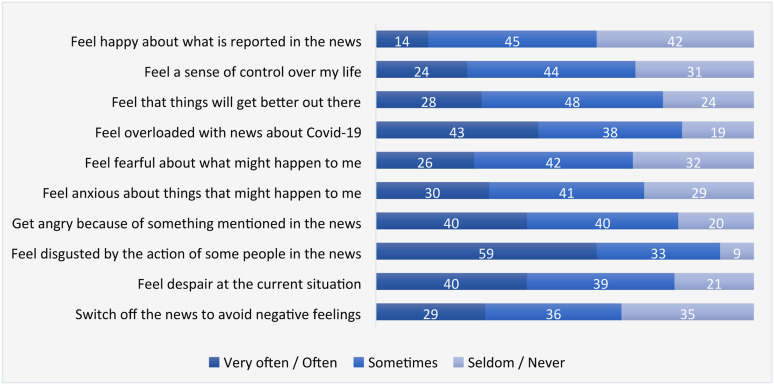
Positive and negative psychological responses to pandemic news (%, n = 2015).

Projecting these into the general population, our survey shows that a large majority of the UK population experienced this overload of bad news, with two-thirds agreeing that “news about Covid-19 is rather repetitive” and more than half (53%) agreeing “there is not enough news around the good things that happen during Covid-19” (Figure 1). In addition, 81% sometimes, often or very often felt “overloaded with Covid-19 news in the past 12 months” (Figure 2).

The range of emotions that emerged out of this “barbed wire” was wide. Throughout the interviews, we found that despair and anxiety were the most frequently mentioned responses to Covid-19 news. To a lesser extent, some interviewees reported instances when they “got mad” or “felt sick” of the behaviours of some people in the news – from the ordinary people who refused to wear masks in a supermarket, to the powerful few who acted against pandemic standards (e.g. the lockdown rule breaking of Dominic Cummings, the former senior advisor to Prime Minister; the way Donald Trump was denying Covid or spreading misinformation about its treatment). In the survey (Figure 2), a large majority of the public said they at least sometimes felt “despair at the current situation” (79%), “anxious about things that might happen to me” (71%) and “fearful about what might happen to me” (68%). However, moral outrage came on top with 92% having at least sometimes felt “disgusted by the action of some people in the news” and 80% “angry because of something mentioned in the news”. Overall, only 25% of respondents in our survey agreed – while 31% disagreed – that “news about Covid-19 generally made my pandemic experience better” (Figure 1).

This manifested in a range of symptoms that were laid bare in interviewees’ accounts, including obsession by a “bleak outlook on the future” (Rohan), sleep deprivation (Helen), “not wanting to go outside” (Nigel), various alterations of regular activities and even the inability to function normally. One of the most sober accounts came from Chloe, who went through to a slump period that saw her unable to get out of bed:

In the first lockdown, watching the news was really negative for me, and it did not help in any way, shape or form … I spent three or four months reading the news and then it got to a point where I just I didn't go out anymore. I didn't walk the dog every day. I stayed in bed, I wasn't eating properly, I went into a slump.

Some had to resort to mental medication. Nina, for example, took the antidepressant Fluoxetine for the first time in her life, to calm herself from the stresses that she attributed substantially to her “focusing so much on the Covid-19 situation on the news”.

Before moving on, it should be noted that such personal accounts cannot be attributed solely to the influx of negative news. In fact, they cannot be removed from the context of the stress, turmoil and uncertainty caused by the pandemic. In such times, there was evidence that even positive developments could become a cause of ill mental health. One respondent, for instance, described how Boris Johnson’s announcement of the roadmap to the new normal a few weeks before the interview had a “massive, massive, massive, massive” effect on her anxiety:

I remember I got halfway down the article to the one line that basically says social distancing will just become non-existent. I started to have a mini panic attack because, after the past year of not being near anyone or not hugging my parents and my grandparents, [it was scary to think] suddenly in a few months’ time I would be able to do that … And I started crying. I was worried about it, and then I saw things like Leeds festival coming … I can't even describe how much anxiety I had and how scared I was … just terrified of the fact that we're going back to square one. (Rebecca)

Regardless of what news content causes mental distresses and disorders, it was clear, expectedly, that they led periods of total or partial news avoidances both between and within phases of the up-down-up cycle. Our interviews reveal evidence of audiences adopting three major strategies of avoidance that have been identified in the literature. At a light level, some adopted a *news dosing* strategy (Groot-Kormelink and Klein-Gunnewiek, 2021), i.e., controlling the amount of news they take in by, for example, fixing it to certain times of the day and/or turning off news app notifications. At a heavier level of avoidance was *a media substitutio*n approach – i.e. limiting their exposure to news content by seeking non-news media alternatives [Newman et al., 2020]. The most frequently cited strategy was *news resistance* (Woodstock, 2014), where users made deliberate attempts to shut their world from the news altogether. Our interviewees, including some avid news users, reported avoiding the news for periods of a few days to several months as, in one participant’s words, “a mental break” from the stress of “looking at that kind of thing every day”. Regardless of the length of switch-offs, participants did feel that avoidance helped them build resilience in the face of calamities, especially to avoid being knocked down by “doom and gloom” thoughts. Greg, for example, found that less news made him “feel more positive about the situation because I’m not bogged down by the stats around cases and deaths”. Meanwhile, Chloe “completely turned all of my social media apps off … for the (Christmas) week” to find that “I’ve got time … and I can breathe” [authors’ emphasis]. Our survey confirmed the strong prevalence of news resistance: two-thirds of the respondents at least sometimes “switched off the news to avoid negative feelings”, with three in ten doing so often or very often (Figure 2).

To gain a deeper quantitative insight, we conducted a regression analysis (Table 1) for news resistance on the frequencies of the aforementioned mental responses – fear, anxiety, disgust, anger, despair and the cognitive information overload – with statistical control for the key demographics of age and gender. We found that these variables together explain about 37% (*p* < 0.001) of the total variance in pandemic news resistance. Individually, all but one of the six feelings – anxiety (coefficient = −0.06, *p* = 0.07) – contributed significantly to that resistance. Of the five significant contributors, interestingly, disgust was a negative one – the more often people felt disgusted by the action of people in the news, the less often they resisted the news during the pandemic. This suggests that even in the face of psychological distresses, news audiences would, out of moral outrage, still keep up with the news to monitor the deeds of those they thought were not behaving properly in relation to the pandemic situation. Age and gender were statistically significant contributors to news resistance in their own right, with females and younger people showing higher frequencies of pandemic news switch-off.

**Table 1.** Relationship between news-induced negative feelings and news resistance during the pandemic.

	Coefficient
*Feel fearful about what might happen to me*	0.07*
*Feel anxious about things that might happen to me*	−0.06
*Get angry because of something mentioned in the news*	0.11***
*Feel disgusted by the action of some people in the news*	−0.60*
*Feel overloaded with news about Covid-19*	0.47***
*Feel despair at the current situation*	0.18***
*Gender (0 = male)*	0.16***
*Age (years)*	−0.01***
Adjusted R-squared	0.37***

n = 2014; F (8, 2005) = 146.5.

### Positive effects of pandemic news on psychological wellbeing (RQ2)

Although feelings about the negative effects of Covid-19 news on public mood were overwhelming, it is important not to lose sight of the fact that there were positive perceptions. As noted, uncertain and unsettling times like the pandemic prompt people to look out to things that are near and dear to their heart – at least to assess the situation, to know what to do, and to have a sense of security that they are on top of the situation. This is where the positive values of news – particularly its reassurance effect – comes in. As Nigel put it: “I don’t know whether they would even have helped me prepare because nothing really helps prepare you for this, but I think knowing was a little bit more, better than just not being aware.” For some, the simple fact of having access to news was a reassurance. Hannah, for example, said that the news has definitely made her pandemic experience better because “knowing what is happening around me and seeing the trends” made her feel in control of the situation, rather than “just imagining scenarios in my head”. As she explained:

Even the slightest small news about the pensioners who got their first jabs, the planning for holiday … gives me a sense of safety. Not safety – I should say it gives me a sense of being in control.

In our survey, there was an equal divide in responses to “news about Covid-19 helps to clear my thinking” (30% agreeing and 30% disagreeing). However, the majority (53%) agreed – and 9% disagreed – that “news about Covid-19 reassures me that I’ve been doing the right thing” (Figure 1). Further, 69% at least sometimes felt “a sense of control over my life” from news consumption (Figure 2).

Some in-depth interviewees found other positives than cognitive reassurance, particularly happiness and hope, from the news they consumed. This was increasingly the case when news about the rollout of vaccines, new treatments and other things became more prevalent. Such “cheerful news”, in an interviewee’s words, “obviously got a bit of a psychological emotional effect on all of us” because it provided “the hope (that) perhaps there's a light at the end of the tunnel” (Mandy). When we brought these into the survey, six in ten sometimes/often/very often felt “happy about what is reported in the news” and three-quarters (76%) at least sometimes felt from news that “things would get better out there” (Figure 2).

Did positive emotions help reduce news avoidance? Our regression of the frequency of news resistance on the frequencies of feeling happiness, hope and a sense of control, with control for age and gender, provided a firm yes to that question. As seen in Table 2, the model explains 13% of the variance in news avoidance (*p* < 0.001). The news-induced feelings of hope and happiness were significantly negative determinants of news resistance. The “sense of control over my life” was also negative but not statistically significant. In other words, there was enough evidence to say that the more frequently people felt positive from pandemic news, the less frequently they abandoned it. Note that the effects of age and gender remain unchanged from those in the previous model for negative emotions.

**Table 2.** Relationship between news-induced positive feelings and news resistance during the pandemic.

	Coefficient
*Feel happy about what is reported in the news*	−0.15***
*Feel that things will get better out there*	−0.07*
*Feel a sense of control over my life*	−0.02
*Gender (0 = male)*	0.33***
*Age (years)*	−0.02***
Adjusted R-squared	0.13***

n = 2014; F (5, 2008) = 62.

### Perceived alternatives to negative pandemic news (RQ3)

What, then, could the media do to make pandemic news more positive, or at least, less negative? Although in-depth interviewees acknowledged that negativity is unavoidable in the pandemic, many argued that the media could create more positive pandemic news by restraining themselves from old habits. In particular, they saw the abundance of negative Covid-19 news as an exacerbation of the inherent imbalance between positive and negative stories in everyday news. Evident in their accounts is the perception that there were positive stories around the pandemic that the media could but chose not to tell. Christopher, for instance, said that he abandoned TV as his main source because it “keeps repeating the same story (that just pushes) one angle”. For him, this is not unintentional. “The [mainstream] outlets would draw a very grim picture of the whole thing,” he said. “Well, the cases are rising, but how often do we hear about people recovering?”

When participants were asked to recommend ways to make pandemic news more positive, their perceptions of what is positive vary from one to the next. Some might strike hard at the very pillars of journalism, such as the following suggestion that the media should be less critical and deliver a “positive spin” on certain things:

The media have been quite critical of public bodies … They've been very critical about the NHS, … about the reporting of numbers, … about the vaccine. I think they could definitely put a more of a positive spin on it, but (they’ve chosen to be) more negative. (Toby)

Most recommendations, however, fall into two categories. First, there was a strong call for *more emphasis on events that lift the public mood*. This is different from asking journalists to put a “positive spin” on events, facts and figures: it means bringing to light the positive things that have come from the pandemic. Chloe, for example, wanted to see the benefits that lockdowns bring. One example she did not “see reported much in the UK is the environmental benefits that we've had”. Others shared the above Christopher’s demand for positive people-centred stories:

I'd like to see more stories regarding people beating Covid-19. Because I think that people have had enough with the negatives, they need hope to know that people are actually recovering from it. (Nigel)

I remember watching and reading one story about a volunteer at a vaccine centre. I didn't look out for it, as that was not the reason I logged in, but I read it and thought ‘oh, that was nice to hear about.’ There have to be hundreds, maybe thousands, of these volunteers but we're not hearing about. It would be nice to hear a couple more of their stories, like ‘a day in the life of’ stories could be nice. (Greg)

One of the most oft-mentioned examples was stories about the 100-year-old Sir Tom Moore’s effort to raise millions of pounds for NHS charities:

When Captain Tom's story came out it was … a very positive story and literally a ray of hope for us. (Hannah)

I think the more positive news you get the more you can cope with it … Positive stories do help. Like Captain Tom, that boosted everyone's morale, didn't it? I think stories like that make people think that whatever was happening with the virus and with lockdown, (everyone) could do something positive. (Irene)

In line with this, nearly half (48%) of survey respondents agreed – and only 15% disagreed – that they “would like to see more news about those who deal well with pandemic challenges” (Figure 1).

Second, there was a desire to see *a shift from an excessive focus on Covid-19 problems to more attention to how people overcome these problems*. Even simple advice on how to cope with the situation could help. Christine – who, “at one point in the second lockdown, … just felt like losing the will to live a little bit” – commented on how some advice in the news empowered her to escape depression:

They give you good ideas on what to do. ‘Make sure you get outside’ and things like that, so I have watched that kind of news. And I took on board some of the things that people have said to do.

Beyond lifestyle advice, others called for something that resemble the core values of what has come to be known as constructive, solutions-oriented journalism (SOJO), a journalism practice that breaks from traditional focus on problems in society to a balance between problems and solutions to problems (Haagerup, 2017). Typical of this is Davide’s call for the news to focus on what governments and other authorities are doing to find a way out of the pandemic:

If there are problems somewhere, what's happening? What's the way out? So, the NHS is overwhelmed what's the way out? How do we build a new hospital? Are we putting in applications into build new hospitals or we're recruiting more staff? … So, it might be bad news now, but is there anything beyond that … to put a smile on our faces … as well as the facts?

## Discussion and conclusion

This research has shown a multifaceted picture of the functions and impacts of Covid-19 news in the daily life of a pandemic-wounded public. On the one hand, people relied heavily on the news as a lifeline to keep themselves informed, assured and, where possible, empowered in the face of extreme uncertainties. On the other, they found an abundance of repetitive bad news that led them to experiencing, intensively or extensively, many mental and emotional wellbeing problems. Inherent in the search for information utilities in pandemic news – primarily for personalised surveillance purposes – is the ambivalence between anxiety and security, with the motive to seek information being characterized by deep ambiguity, uncertainty and, hence, distress. By combining two rich sets of qualitative and quantitative data, this research adds depth to previous research that has found how news emphasizing negativity can lead to disillusion and disinterest in matters of civic interest, and, ultimately, provoke a civic disengagement with social and political matters (Moeller, 1999; Kinnick et al., 1996).

Perhaps one of the most telling parts of the data are the sober personal testimonies on how the adverse impacts of news consumption manifest the way people altered their daily activities and routines during the pandemic (e.g., sleep deprivation, no desire to go out, bedridden depression, medication and, of course, news avoidance). Such psychological discomfort and disorders laid bare the very real and dramatic impacts of negative news on the fabric of society. They speak to a larger issue often expressed about the mental and emotional effects of the 24-h news cycle of unfinished stories. In this cycle, news outlets must keep reporting, even with little new information, while the public gets increasingly bored, fatigued, desensitised, stressed and sometimes despaired (Lewis and Cushion, 2009; Saltzis, 2012). Our mixed-method data show that most users, being ceaselessly exposed to a prolonged repetition of bad news around Covid-19, would sooner or later reach a point of informational saturation, emotional drain and mental distress. Amidst a general climate of uncertainty and anxiety, we saw fear, anger, disgust, despair and cognitive overload emerging both as the most prevalent psychological responses to pandemic news content and as direct triggers of the alteration of regular daily activities, including the partial or total abandonment of news. The regression data show a clear contribution of these news-induced emotions to news resistance, especially among younger females. In short, news resistance – and the lesser forms of news avoidance (news dosing and media substitution) – could be seen an essential part of living in a precarious and threatening situation, serving as a resilience-building strategy against psychological disturbances.

It would be a critical mistake, however, to think of pandemic news exclusively in terms of its negative psychological effects. This study breaks from previous research into pandemic news consumption by paying close attention to its positive aspects. It was clear from both the interview and survey data that news was at least an occasional source of reassurance, happiness and hope for the majority of audiences during the pandemic. The regression analysis further shows that such positive psychological experiences did help to prevent people from tuning out the news. This echoes the argument by (Groot-Kormelink and Klein-Gunnewiek, 2021: 8) that “news is simultaneously a source of fear and comfort”: during a dark time like this pandemic, it can expose audiences to the danger of mental erosion at the same time as reassuring and empowering them.

This study thus highlights the urgent need for journalists and media executives to take lessons from their heavily negative pandemic coverage to reconsider the important but often overlooked function of news as a force to inspire, motivate and empower the public. This invites a fundamental shift from traditional occupational ideologies that prioritise negative news – i.e. the type of “if it bleeds, it leads” mindset – to a balance of the negative and the positive. Of course, the pursuit of bad news to inform and enlighten the public is integral to journalism’s watchdog role and its professional reputation. However, over the years this conditioned journalists towards an overemphasis of reporting the negative at the expense of the positive. In this particular case, while the pandemic’s unprecedented disruptive and destructive scale might make it hard for journalists to be positive in news reporting, the suggestions from our interviewees did point to ample space for improvement. Audiences do not always speak the same language as journalists but, as the interviews demonstrate, they did see many ways in which pandemic news can be made more positive.

In relation to this, Beiler and Krüger, 2018 remind us of the normative underpinning of the role of the press in democracies, going back to ideas around social responsibility of journalism and a resulting moral commitment for the news to embrace the needs of society. Here, the media can provide a fundamental public value to democratic societies, if they are understood to “not only entertain and to inform, but to provide a positive contribution to society” as a normative aim (p. 170). “Positive” in this sense can be understood as allowing orientation in a complex world, mobilizing civic participation and, crucially, not overwhelming users with negative problems-focussed news coverage. Perhaps the pandemic news experience of the UK public could be a call for journalists to shift to a more balanced approach that incorporates more positive, productive and socially shared elements. Although such balanced models – e.g. public journalism, peace journalism and solutions journalism – are yet to see a breakthrough in market-oriented journalism, they have shown promises as forms of “mental hygiene”, morale boosters and action stimulators (McIntyre, 2019). Embracing such practices – to put smiles on audiences’ faces, to recite an interviewee – would help reduce news avoidance and ensure that decades of declining audiences do not culminate into an existential crisis for journalism.
